# Cardiac Autonomic Adjustments During Baroreflex Test in Obese and Non-Obese Preadolescents

**DOI:** 10.5935/abc.20160040

**Published:** 2016-04

**Authors:** Mário Augusto Paschoal, Aline Carnio Brunelli, Gabriela Midori Tamaki, Sofia Serafim Magela

**Affiliations:** Pontifícia Universidade Católica de Campinas, PUC-Campinas, Campinas, SP - Brazil

**Keywords:** Heart Rate/physiology, Autonomic Nervous System/physiopathology, Obesity, Barorreflex, Physical Exertion, Adolescents

## Abstract

**Background:**

Recent studies have shown changes in cardiac autonomic control of obese preadolescents.

**Objective:**

To assess the heart rate responses and cardiac autonomic modulation of obese preadolescents during constant expiratory effort.

**Methods:**

This study assessed 10 obese and 10 non-obese preadolescents aged 9 to 12 years. The body mass index of the obese group was between the 95th and 97th percentiles of the CDC National Center for Health Statistics growth charts, while that of the non-obese group, between the 5th and 85th percentiles. Initially, they underwent anthropometric and clinical assessment, and their maximum expiratory pressures were obtained. Then, the preadolescents underwent a constant expiratory effort of 70% of their maximum expiratory pressure for 20 seconds, with heart rate measurement 5 minutes before, during and 5 minutes after it. Heart rate variability (HRV) and heart rate values were analyzed by use of a software.

**Results:**

The HRV did not differ when compared before and after the constant expiratory effort intra- and intergroup. The heart rate values differed (p < 0.05) during the effort, being the total variation in non-obese preadolescents of 18.5 ± 1.5 bpm, and in obese, of 12.2 ± 1.3 bpm.

**Conclusion:**

The cardiac autonomic modulation did not differ between the groups when comparing before and after the constant expiratory effort. However, the obese group showed lower cardiovascular response to baroreceptor stimuli during the effort, suggesting lower autonomic baroreflex sensitivity.

## Introduction

Expiratory efforts maintained for a certain time against a constant pressure can simulate the autonomic function test, known as Valsalva maneuver.

The Valsalva maneuver was named after Antônio Maria Valsalva, who described it for the first time in 1704, and used it to expel mucopurulent secretion from the middle ear to the nasopharynx. Many years later, it was shown to cause autonomic cardiac and vascular oscillations intermediated by the baroreceptor system.^1,2^

Since then, that maneuver began to be used as a non-invasive autonomic cardiac function test, being standardized as an expiratory effort equivalent to 40 cmH_2_O, maintained for 15 to 20 seconds.^3^ That test usually assesses heart rate (HR) and systemic blood pressure (BP) behavior in response to a stimulus that sensitizes baroreceptors, chemoreceptors and cardiopulmonary receptors, through overload of the cardiovascular system caused by the Valsalva maneuver.^4^

The Valsalva maneuver can be applied to assess baroreflex-dependent cardiocirculatory responses in several situations, such as in ill and healthy individuals, in pre- and post-physical training periods, or in comparative studies of autonomic cardiac modulation between groups of athletes and sedentary individuals.^5,6^

However, it is rarely applied to children and preadolescents, because, according to some authors, the expiratory pressure exerted can be excessive. That population is believed to have difficulty correctly exerting the expiratory pressures and maintaining them for the established time, therefore encouraging the development of studies on that issue.^1^

Several studies have suggested the existence of cardiac dysautonomia in obese children and preadolescents,^7,8^ mainly the morbid obese ones. This study aimed at assessing the HR responses and cardiac autonomic modulation of obese preadolescents during a constant expiratory effort.

The tested hypothesis was that such functional test, by provoking an autonomic cardiorespiratory reflex response, could also reveal the presence of dysautonomia in obese children.

## Methods

This cross-sectional study was approved by the Ethics Committee of Research in Human Beings of the Life Sciences Center of the Pontifícia Universidade Católica of Campinas (protocol 0298/11).

### Study sample

This study selected 20 preadolescents aged 9 to 12 years, who were divided into two groups of 10 individuals each, as follows: obese sedentary (OB); and non-obese (NO). This sample was selected by using the convenience sampling technique.

The inclusion criteria were as follows: no regular physical activity practice; no medication that interfered on the data studied; and no changes on clinical examination. In addition, the 10 obese preadolescents had to have body mass index (BMI) values between the 95^th^ and 97^th^ percentiles of the CDC National Center for Health Statistics growth charts,^9^ while the 10 non-obese ones, between the 5^th^ and 85^th^ percentiles.

### Anthropometric assessment

The anthropometric data assessed were body weight, height, BMI, perimeters of body segments (arm, forearm, thigh, leg and abdomen) and local fat (subscapular, suprailiac, triceps and abdominal).

To measure body weight, individuals should be barefoot and positioned on a pre-calibrated Filizola^®^ scale graded in 100-g units. Height was taken on that same device, using a metallic rod graded in centimeters, with the volunteer in the standing position, facing back the metallic rod, which should be positioned above the head.

In addition, the body perimeters were measured by using a flexible measuring tape, and the skinfolds, by using a scientific caliper (Premier Cescorf^®^, Porto Alegre, RS, Brazil) graded in millimeters.

### Clinical assessment

The clinical assessment consisted of a brief anamnesis to confirm the sedentary lifestyle. Heart rate and BP were recorded. For BP measurement, a standard aneroid sphygmomanometer (Wan Med^®^, São Paulo, SP, Brazil) was used, with cuffs adequate for the participants' arm circumference.

In addition, cardiac and pulmonary auscultations were performed in all participants with a stethoscope (Littmann Classic II^®^, USA), according to the techniques widely described in the literature.

### Obtaining maximal expiratory pressure

Aiming at selecting the expiratory pressure to be used to assess expiratory resistance to stimulate the baroreceptor reflex, the maximal expiratory pressure (PEmax) of each participant was obtained. For that, an M-120 analogical manovacuometer (Global Med^®^, Minas Gerais, Brazil), graded in cmH_2_O, was used. Before that assessment, all participants were instructed on the maneuver to be performed.

The participants were then asked to sit, using a nose clip to prevent air from escaping. They were then instructed to inspire deeply through the mouth, and, right after, to suddenly expire as strongly as possible against the manovacuometer's resistance.

That maneuver was performed three times, at 1-minute intervals. At the end, the nose clip was withdrawn, and the participant rested for 5 minutes. The highest measure of the three interventions (PEmax) was selected to serve as basis for calculating the effort the participant would have to perform for the expiratory resistance maneuver. That effort should correspond to 70% of the participant's PEmax. That percentage was based on previous calculations performed in pilot studies showing that, at that expiratory effort intensity, cardiocirculatory responses are not impaired, and participants can maintain the expiratory pressure with low oscillation for the 20 seconds of the test.

Participants began to be prepared for the expiratory effort test 5 minutes after the last PEmax measurement, when a belt was fixed to their chest to register their heartbeats with a frequency meter (Polar S180^®^, Kempele, Finland). That device has a belt with an elastic system tied to the back and a wristwatch, with which heartbeats can be measured. Later, the heartbeats recorded were entered to a computer, with an IR interface, and by use of the Polar Precision Performance^®^ software (Kempele, Finland), the HR values analyzed during the maneuver, as well as the HR variability (HRV) index, could be calculated.

### Expiratory effort test performance and baroreceptor reflex stimulus

After the 5-minute rest, the participant initiated the expiratory effort test, using a nose clip. The participant inspired deeply through the mouth, and then performed the predetermined expiratory effort (70% of PEmax), which should be continuous for 20 seconds, maintaining the expiratory pressure. During the effort, to facilitate its control, the participant was instructed to read on the manovacuometer display the pressure value, highlighted in red, that should be achieved and maintained.

The tests were considered valid when the highest pressure oscillation during 20 seconds was 5 cmH_2_O. It is worth noting that data were collected under controlled conditions (temperature of 23°C and at the same day times) to avoid the circadian influences of HR on autonomic modulation.

Later, participants remained in the dorsal decubitus position for 5 minutes more, resting, to record the heartbeats of the post-test condition.

### Data collection for HR variability analysis

Before and after the expiratory maneuver, heartbeats were recorded for 5 minutes, so that the autonomic balance would be compared on those two moments. The objective was to know whether, after performing the expiratory effort, the OB group would have more difficulty than the NO group to return to the cardiac autonomic modulation pattern of before the effort.

The HRV analysis involved the time and frequency domains. For the time domain, the following indices were selected according to the Task Force:^10^

- iRR: RR intervals between each normal heartbeat;
- pNN50: percentage of adjacent iRR values greater than 50ms. It represents the parasympathetic influences on the iRR, because the actions controlled by the parasympathetic nervous system are faster than those modulated by the sympathetic nervous system; when greater than 50ms and frequent, they can mean greater vagal interference in heart functioning;
- rMSSD: square root of the sum of the square of the differences between iRR. Similarly to pNN50, rMSSD expresses interferences of the parasympathetic nervous system in the heart, and the higher its value, the greater the vagal action on the heart.
  For HRV analysis in the frequency domain, the following indices were selected:
- LF NU: low frequency component (0.04 to 0.15 Hz), whose values express cardiac sympathetic tonus, although some authors report a certain vagal influence on those values. In the present study, that HRV parameter was normalized (normalized units - NU), according to the Task Force,^10^ and presented as percentages. The values therefore calculated express the percentage influence of the sympathetic component on cardiac autonomic modulation on the occasion of heartbeat recording, considering the total potency of the spectrum after eliminating the influence of the values of the very low frequency (VLF) component, because they have less influence on short-term records;
- HF NU: high frequency component (0.15 to 0.4 Hz), whose values express cardiac parasympathetic tonus. Those values were also normalized according to the Task Force.^10^

### Collection of HR values and calculation of the delta HR 0 to 10 seconds and 10 to 20 seconds during forced expiration

The HR values were recorded during the expiratory maneuver at an intensity of 70% of the PEmax and analyzed in the computer. The Polar Precision Performance^®^ software (Kempele, Finland) presented graphically all the HR behavior before, during and after the maneuver. The value immediately before beginning the expiratory maneuver was recorded and compared with those of the times 0-10 seconds and 10-20 seconds of the maneuver. From those values, all HR variations were calculated.

The period of 20 seconds refers to the exact duration of the expiratory effort during the maneuver and represents, through HR elevation in the period, the interference of the baroreceptor reflex. Greater HR elevations may suggest greater sensitivity to the baroreflex, and, thus, good heart response to the autonomic nervous system stimulus resulting from the expiratory maneuver.

### Data analysis and statistical approach

Statistical analysis was performed with the Graph Pad Prism 4.0^®^ (San Diego, USA) software. The anthropometric and clinical data were shown in tables as means and standard deviations. The Shapiro-Wilk test was used to assess data normality, and, because of their normal distribution, Student *t* test was used to show the differences (p < 0.05) between the groups.

The Shapiro-Wilk test was used to assess the distribution of HRV data, and, because of their non-normal distribution, the nonparametric Mann-Whitney test was used to compare the indices before and after the expiratory maneuver. To compare the HR values (pretest vs. 10 seconds and 10 seconds vs. 20 seconds during the maneuver), the Kruskal-Wallis test and Dunn's post-test were used. The significance level adopted was p < 0.05.

## Results

Table 1 shows the anthropometric data of all participants, while Table 2, their clinical data. The weigh and BMI values were higher in the OB group, which was expected and is part of the study's inclusion criteria.

**Table 1:** Anthropometric data

**Anthropometric data**	**Non-obese (n = 10)**	**Obese (n = 10)**	**p Value**
Age, years	9.6 ± 0.5	9.5 ± 0.5	> 0.99
Weight, kg	38.8 ± 4.9	51.8 ± 4.8	0.0002
Height, m	1.4 ± 0.07	1.4 ± 0.06	0.74
BMI, kg/m2	18.5 ± 1.9	24.5 ± 2.0	< 0.0001
Arm, cm	22.3 ± 1.6	26.9 ± 1.5	0.0001
Forearm, cm	19.1 ± 1.2	21.8 ± 1.0	0.0002
Thigh, cm	41.5 ± 3.8	47.2 ± 4.4	0.015
Leg, cm	28.8 ± 1.3	33.3 ± 2.5	0.0004
Abdomen, cm	65.5 ± 6.4	77.7 ± 6.1	0.0021
**Skinfolds, mm**			
Subscapular	15.5 ± 7.3	24.4 ± 7.6	0.0362
Triceps	21.1 ± 8.2	31.1 ± 6.0	0.0098
Abdominal	27.1 ± 10.0	41.6 ± 7.0	0.0055
Suprailiac	34.0 ± 15.8	51.6 ± 8.6	0.0066

Data presented as mean ± standard deviation. BMI: body mass index.

**Table 2:** Clinical data

**Dados clínicos**	**Non-obese (n = 10)**	**Obese (n = 10)**	**p Value**
HR, bpm	93.4 ± 13.5	86.4 ± 9.6	0.42
Systolic BP, mm Hg	106.6 ± 8.6	100.0 ± 7.2	0.65
Diastolic BP, mm Hg	56.6 ± 5.0	60.0± 7.0	0.07
PEmax, cmH2O	80.7 ± 20.5	77.3 ± 15.9	0.43
70% PEmax, cmH2O	56.4 ± 14.1	54.1 ± 11.1	0.43

Data presented as mean ± standard deviation. HR: heart rate; BP: blood pressure; PEmax: maximal expiratory pressure.

All body segments and skinfolds assessed differed significantly (p < 0.05) between groups, and the OB group had always the greatest values.

Despite obesity, no clinical differences in HR and systolic and diastolic BP were observed between the groups. In addition, the clinical parameters were within the normal range.

Similarly to the clinical data, the HRV indices did not differ in the pre- and post-expiratory maneuver conditions (Table 3). This shows that, after the effort, the HRV values expressing the cardiac autonomic modulation returned to their pre-effort values.

**Table 3:** Mean values of heart rate variability (HRV) indices before and after the expiratory maneuver

**HRV Indices**	**Before**			**After**		
	**Non-obese (n = 10)**	**Obese (n = 10)**	**p Value**	**Non-obese (n = 10)**	**Obese (n = 10)**	**p Value**
RR intervals, ms	664.0	655.0	0.73	673.0	678.0	0.5
pNN50, %	6.6	9.5	0.79	6.9	11.9	0.4
rMSSD, ms	40.5	52.8	0.66	43.0	50.0	0.2
LF, NU	58.2	37.2	0.38	59.9	47.7	0.2
HF, NU	41.7	62.7	0.38	40.0	52.2	0.2

pNN50: percentage of adjacent RR intervals greater than 50ms; rMSSD: square root of the sum of the square of the differences between RR intervals; LF: low frequency component; HF: high frequency component; NU: normalized unit.

**Figure 1:**
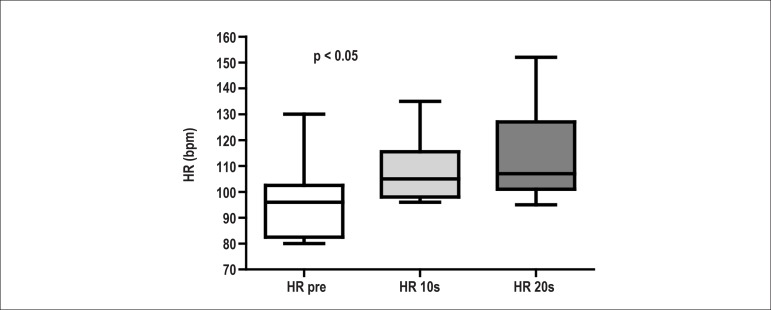
Median values, first and third quartiles, and extreme heart rate (HR) values obtained right before the beginning of the expiratory effort (HR pre), after 10 seconds from the beginning of the expiratory effort (HR 10s) and on the 20th second of the expiratory effort (HR 20s), with 70% of the maximal expiratory pressure, obtained in the non-obese preadolescent group. Kruskall-Wallis test. p = 0.0432 - significant difference.

Figure 1 shows, as boxplots, the median values of the first and third quartiles, and the extreme HR values obtained in the NO group before and during the expiratory effort (10 and 20 seconds). The HR showed a trend towards elevation from the beginning to the end of the expiratory effort, characterizing the normal HR response to the baroreceptor reflex in the NO group.

**Figure 2:**
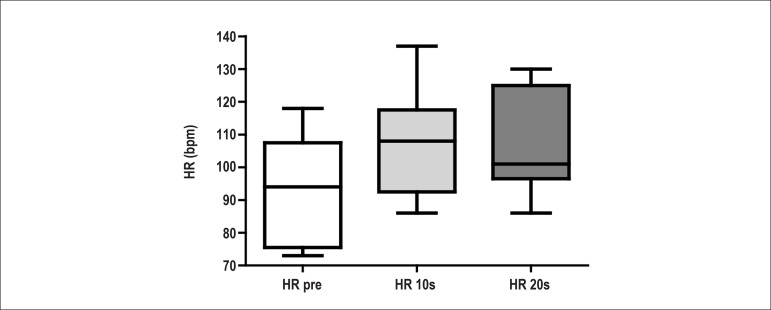
Median values, first and third quartiles, and extreme heart rate (HR) values obtained right before the beginning of the expiratory effort (HR pre), after 10 seconds from the beginning of the expiratory effort (HR 10s) and on the 20th second of the expiratory effort (HR 20s), with 70% of the maximal expiratory pressure, obtained in the obese preadolescent group. Kruskall-Wallis test; p = 0.1332 - no significant difference.

Under the same conditions, the OB group did not show the same HR behavior (Figure 2). The HR value increased up to the 10^th^ second of the expiratory effort (HR pre compared to HR at 10 seconds); however, the HR value did not increase from the 10^th^ to the 20^th^ second of the expiratory effort.

## Discussion

In addition to showing that the expiratory pressure to be applied during the expiratory effort similar to the Valsalva maneuver can be individually calculated, the present study aimed at assessing whether the magnitude of the cardiovascular response to the baroreceptor reflex stimulus of obese preadolescents would differ from that of the healthy control group.

The major findings of the present study were: an expiratory effort calculated as 70% of the PEmax and maintained for 20 seconds, although slightly greater than that proposed in other studies,^1^ can be applied in functional tests to assess cardiovascular responses to stimuli promoted by baroreceptors; the cardiac autonomic modulation, which was similar in the OB and NO groups before the expiratory effort, returned rapidly to its characteristic after the effort; the OB group did not show the same magnitude of the HR response stimulated by baroreceptors, unlike that of the NO group, this being the most relevant finding of this study.

A reduction in the baroreflex response has also been reported in children and preadolescents by Dangardt et al.^11^ and Lazarova et al.^12^ The analysis of the baroreceptor reflex is important to assess the cardiac baroreflex activity, because it incorporates both the sympathetic and parasympathetic afferent and efferent branches; therefore, its assessment could be more sensitive than HRV to identify cardiac autonomic dysfunction in children.^11,13^

The analysis began with anthropometric and clinical data, which could raise questions whether they could have interfered in the above results, and showed that the mean values of the body segments and skinfolds of the OB group were significantly greater, as expected.

Those values certainly contributed to the higher body weight of the obese individuals and their inclusion in this study. It is worth noting that the greater abdominal perimeter was confirmed in the obese individuals. That body region measure has clinical relevance because it correlates with an increased risk for cardiovascular diseases, such as coronary artery disease,^14^ acute myocardial infarction,^15^ and diabetes,^16^ and can even interfere with cardiac autonomic modulation.^17-20^

The values of systolic and diastolic BP and of HR did not differ between the groups, suggesting that obesity has no effect on them, as reported by some studies.^15,17^ However, that is a controversial issue, because some studies^14,21^ have reported higher BP and HR values in obese preadolescents, including increased vascular stiffness of their carotid artery.^22^

Briefly, it seems that the significant effect of obesity on those clinical data is not simple, and some factors, such as genetic inheritance, obesity duration and presence or absence of sedentary lifestyle, have been suggested to be related and require further investigation.

In addition, the PEmax values did not differ between the groups. It is worth noting that, if they did differ, they could account for the difference in the HR data obtained during the expiratory effort, as shown in another study conducted by our team.^1^

Regarding the HRV indices in the time and frequency domains, no significant difference was identified between the groups (before or after the expiratory effort, when the heartbeats were recorded).

However, unlike our results, some studies^7,17^ have shown differences in the cardiac autonomic modulation between obese and non-obese preadolescents, tending towards a reduction in vagal activity and an increase of the cardiac sympathetic tonus in the obese ones. Other authors have suggested that, in that population, dysautonomia relates to a decrease in the sympathetic and parasympathetic activity.^23^

The lack of difference in the HRV indices obtained in this study, in addition to suggesting normality of the autonomic nervous system at rest in both groups, contributes to prevent that possible changes in the OB group could be held responsible for the differences in HR responses, which were documented during the baroreceptor reflex stimulation.

The most important finding of this study occurred during the expiratory effort, which triggered the baroreceptor reflex. Analyzing the HR behavior on the occasions of pre-effort rest and at the 10^th^ and 20^th^ seconds of the expiratory test, the OB group did not show the same HR response pattern of the NO group, suggesting cardiac autonomic dysfunction in that group.

During expiratory efforts sustained for a certain time, HR elevation usually occurs, as reported in studies using the Valsalva maneuver. In those studies, the HR elevation that occurs from time 0 (beginning of effort) to 10 seconds of the effort is vagus-dependent, that is, there is vagal release, which determines a rapid HR elevation; the HR elevation in the final 10 seconds occurs in response to arteriolar sympathetic activation.^1,3,8^ However, some authors consider it difficult, from the methodological viewpoint, to separate the HR response during the baroreflex stimulus promoted by the expiratory effort into times (from 0 to 10 seconds, and from 10 to 20 seconds).^24^

Thus, the OB group, with similar values to those of the NO group at rest, cannot have the same cardiovascular performance when stimulated by use of the baroreceptor reflex.

However, when analyzing HR values obtained during the expiratory effort in adults, because parameters in adolescents are scarce, the HR differences from the beginning of the expiratory effort to the end of the 20^th^ second showed that obese individuals had a mean 12.2-beat elevation while non-obese had an 18.5-beat elevation. According to Hohnloser and Klingenheben,^3^ that would represent normal cardiac response of non-obese individuals, and borderline (upper limit of normality) response of the obese ones.

In addition, according to Hohnloser and Klingenheben,^3^ delta HR values ≥ 15 bpm indicate proper cardiac autonomic response, while delta HR values between 11 and 14 bpm are considered borderline. When delta HR values ≤ 10 bpm, such as those usually found in heart failure, diabetes, post-acute myocardial infarction, and mitral stenosis, the response is considered abnormal.

It is difficult to explain why the OB group did not have the same performance of the NO group, although some studies, such as that by Wieling et al.,^25^ have reported that the functional changes in the reflex loop, responsible for the chronotropic activity regulation that results in insufficient HR elevation during provocative stimuli, could suggest autonomic dysfunction.

According to Rabbia et al.,^8^ obese preadolescents tend to have sympathovagal dysfunction, which hinders the baroreflex control of HR, as evidenced in the present study, and these results are in accordance with those by Dangardt et al.^11^

The small sample size is a limitation of this study. Therefore, further investigation involving a higher number of participants should be conducted to confirm the lower baroreflex responsiveness detected in the OB group.

## Conclusion

The Valsalva maneuver applying a resistance equivalent to 70% of the PEmax can be used in a population of preadolescents to assess the cardiovascular response to the baroreceptor system stimulus.

This study most important finding was that obese preadolescents had lower autonomic baroreflex responsiveness than non-obese ones, because they had lower total HR response during the expiratory effort used to stimulate the baroreceptor reflex.
